# Developing the ethics of implementation research in health

**DOI:** 10.1186/s13012-016-0527-y

**Published:** 2016-12-09

**Authors:** Vijayaprasad Gopichandran, Valerie A. Luyckx, Nikola Biller-Andorno, Amy Fairchild, Jerome Singh, Nhan Tran, Abha Saxena, Pascal Launois, Andreas Reis, Dermot Maher, Mahnaz Vahedi

**Affiliations:** 1 Department of Community Medicine, ESIC Medical College and Postgraduate Institute of Medical Sciences and Research, KK Nagar, Chennai, 600078 India; 2 Institute of Biomedical Ethics and History of Medicine, Center for Medical Humanities, University of Zurich, Winterthurerstrasse 30, CH-8006 Zurich, Switzerland; 3 Associate Dean of Academic Affairs, Texas A & M School of Public Health, College Station, Texas, USA; 4 University of KwaZulu-Natal, Nelson Mandela School of Medicine, 719 Umbilo Road, Durban, 4001 South Africa; 5 Alliance for Health Systems and Policy Research, World Health Organization, 20, Avenue Appia, 1211 Geneva, Switzerland; 6 Global Health Ethics Unit, World Health Organization, 20, Avenue Appia, 1211 Geneva, Switzerland; 7 Special Programme for Research and Training in Tropical Diseases (WHO/TDR), Geneva, Switzerland

## Abstract

Implementation research (IR) is growing in recognition as an important generator of practical knowledge that can be translated into health policy. With its aim to answer questions about how to improve access to interventions that have been shown to work but have not reached many of the people who could benefit from them, IR involves a range of particular ethical considerations that have not yet been comprehensively covered in international guidelines on health research ethics. The fundamental ethical principles governing clinical research apply equally in IR, but the application of these principles may differ depending on the IR question, context, and the nature of the proposed intervention. IR questions cover a broad range of topics that focus on improving health system functioning and improving equitable and just access to effective health care interventions. As such, IR designs are flexible and often innovative, and ethical principles cannot simply be extrapolated from their applications in clinical research. Meaningful engagement with all stakeholders including communities and research participants is a fundamental ethical requirement that cuts across all study phases of IR and links most ethical concerns. Careful modification of the informed consent process may be required in IR to permit study of a needed intervention. The risks associated with IR may be difficult to anticipate and may be very context-specific. The benefits of IR may not accrue to the same groups who participate in the research, therefore justifying the risks versus benefits of IR may be ethically challenging. The expectation that knowledge generated through IR should be rapidly translated into health policy and practice necessitates up-front commitments from decision-makers to sustainability and scalability of effective interventions. Greater awareness of the particular ethical implications of the features of IR is urgently needed to facilitate optimal ethical conduct of IR and uniform ethical review.

## Introduction

Implementation research (IR) is growing in importance and recognition: there is an increasing funding from a range of donors/sponsors for this research area, leading scientific journals have established sections promoting the publication of such research, and it contributes increasingly to the evidence-base used by the World Health Organization (WHO), which promotes, supports, publishes, and evaluates such research [1]. With its aim to answer questions about how to improve access to interventions that have been shown to work but have not reached many of the people who could benefit from them, IR involves a range of particular ethical considerations that have not yet been comprehensively covered in most international guidelines on health research ethics [2, 3]. The draft of the Council for International Organizations of Medical Sciences (CIOMS) guidelines which is currently under revision as well as its commentary does briefly allude to ethical considerations in the conduct of cluster randomized trials (CRT), but thus far there has been no comprehensive discussion or guideline regarding the application of ethical principles in IR in general or in relation to study designs beyond CRTs [4].

In response to the need for more clarity and guidance about the ethical implications throughout the IR process, Special Programme for Research and Training in Tropical Diseases (TDR) and the Global Health Ethics unit in collaboration with the Alliance for Health Policy and Systems Research at the World Health Organization are developing a training tool, the Ethics in Implementation Research Toolkit, as a practical guide for IR researchers and ethics committees to facilitate optimal study design, conduct, and review. The training tool was developed through a consultative process launched in Geneva in which IR experts, philosophers, ethics committee members, and public health practitioners met to identify the ethical issues in IR, define the course content and the format of the training workshops. The tool was further developed through small group work with the support of an expert in adult training methods. The tool has been validated in two pilot workshops in Asia and Africa. The list of experts who contributed to the development of the Toolkit, apart from the authors of this manuscript, can be found in the Acknowledgements section.

This manuscript is constructed around the concepts identified during the development and piloting of the Ethics in Implementation Research Toolkit and aims to highlight the differences in application of ethical principles between clinical and implementation research, and to highlight the current gaps in ethical guidelines for the conduct of IR.

## Background

IR involves increasing the understanding of how to improve access to health products and strategies that are already available and have been shown to work, but remain beyond the reach of many of the people who could benefit from them. IR therefore provides the link between what should happen in theory and what actually happens in practice. It is rooted in the identification of practical problems facing disease control programmes and in finding solutions which improve access to health interventions and lead to better health outcomes. IR addresses different aspects of implementation including social and contextual factors (poverty, environment, culture), the process of implementation (which approach best answers the implementation issue?) or the outcomes of implementation (clinical/process end points). For example, in case of a new vaccine for prevention of dengue, basic science and traditional clinical research address vaccine development and safety and efficacy testing. IR then addresses the questions of accessibility, acceptability, appropriateness, and feasibility in the communities where the vaccine is needed. IR questions, however, are not always related to a clinical disease entity or implementation of a treatment or prevention program. IR also addresses process issues in health care delivery, as well as cost-effectiveness, policy uptake and implementation, health education etc. IR therefore draws on a wide variety of research approaches to address the diverse research questions. The research designs therefore are not restricted to traditional trial designs, but include methods such as participatory action research (PAR),^1^ qualitative design, and effectiveness implementation hybrid designs 2. Flexibility is a great advantage in IR as the research question largely drives the design, the research process is iterative, and the findings at each stage feed back into the design. IR is usually carried out in close collaboration between researchers and disease control programme staff or policy-makers. The costs are generally modest, yet IR has the potential for a large magnifier effect, as effective implementation expands the impact of health interventions delivered by programmes. As a pre-requisite therefore to the design of a successful implementation strategy, the clinical/public health problem must be identified, the epidemiology of the disease/health status must be understood, and a situation analysis must be performed to identify why access is sub-optimal, and what the actual bottlenecks/gaps in care delivery are (not merely presumed), such that interventions can be targeted to reduce these bottlenecks/gaps. In the case of adoption of a successful intervention from one country by another country or scaling-up of interventions from a pilot phase to a wide area, a local situational analysis should be carried out to determine differences and similarities between the communities where an intervention has been successfully implemented and the communities in which the intervention will be tested. IR is relevant when this analysis shows important differences but points to the proposed intervention as the most appropriate strategy, or justifies full scale implementation of the intervention [5]. Awareness of the appropriate application of ethical principles in IR is important in study design and data generation to ensure ethical conduct of IR and to effectively contribute to health system strengthening. In the planning stages, researchers must also be able to effectively communicate their consideration of the ethical principles to research ethics committees, who must also have insight into the adaptations of ethical principles required in IR (as opposed to traditional clinical research) such that protocols are appropriately and fairly reviewed.

### Are there ethical considerations which apply particularly to implementation research?

IR is aimed at identifying the best process to implement and scale-up research evidence, whereas biomedical and clinical research focuses on establishing the evidence. This fundamental difference between clinical and implementation research necessitates a modification in the application of ethical principles in their conduct as highlighted in Table 1.

**Table 1:** Differences between clinical and implementation research which impact application of ethical principles^a^

Domain	Clinical research	Implementation research
Research participants	Individuals	Countries, institutions, communities, and individuals
Informed consent	Informed consent by competent individuals, assent by minors and consent by legally authorized representatives	Consent may be difficult to obtain in cluster randomized trial design. There may be a need for a two level consent—consent for randomization from gatekeepers and consent for participation at the individual level. Sometimes individual consent may not be feasible. However, gatekeeper consent does not replace the need for individual consent. Ethical committee should oversee the informed consent requirement and process
Equipoise	Clinical equipoise	Clinical as well as contextual equipoise (genuine uncertainty that the implementation will work in a new context as well as whether the implementation package will work at all)
Pre-requisites	Understanding of disease pathophysiology Intervention aimed at disease-specific management	Identification of population health needs Understanding relative priority of need for intervention within local context Community engagement to understand community needs, ensure scalability, and sustainability
Research conditions	Generally controlled research environment	Real-life or pragmatic research environment
Research designs	Cross-sectional, case-control studies, Cohort studies, randomized clinical trials	Cluster randomized trials Pragmatic, mixed methods, effectiveness implementation hybrid designs, participatory action research, quasi-experimental design, realist review
Integration within health system	Often, there is no a priori plan for health system integration. Findings of clinical research go through IR before integration into health system	IR has a strong health system strengthening focus. It creates horizontal integration into the health system. There is an ethical imperative for health system integration
Predominant research disciplines	Physiology, genetics, biochemistry, and other basic sciences, epidemiology, clinical medicine	Anthropology Economics Epidemiology Political science Public health Sociology
Control groups	In most epidemiological designs, control groups are required. But some phase 1 clinical trials and observational studies may not require control groups	Having a no intervention control group may not be acceptable. Alternative designs of quasi-experimental studies do not require a control group
Boundary between research and clinical care	This boundary is usually clear, but may be unclear in case of therapeutic misconception especially in cancer trials	Is often unclear, because the intervention is of proven efficacy
Types of research question	Efficacy and safety of a therapeutic strategy in the individual	Operationalization of an intervention in local context Implementation of an intervention in local context prior to scale-up Policy analysis Health system functioning at multiple levels
Anticipated outcomes	Well-defined hypothesis at the beginning of the clinical research. Expected outcomes clearly stated.	Multifaceted holistic impact on health systems functioning with regard to intervention tested. Sometimes outcomes may be unexpected
Risks assumed by:	Mostly, the risks are for the study participants. However, families and communities may also be affected in specific contexts	Usually population level risks. Moreover, the people getting the benefits and people suffering the risks may be different.
Benefits accrued by:	Benefits accrue to the participants, the community. The research finding may be a common good	Individuals, communities, health system, institutions may benefit. The research findings may be common good. The people accruing benefits may be different from those who suffer risks
Generalizability	Generalizability is sometimes possible in multicentric and well sampled studies, however most studies are specific to the target populations.	Generalizability may be limited by contextual factors. However, findings may be generalizable to similar contexts
Social justice implications	Social justice is usually not a primary consideration. However, justice considerations are required in selection of research participants. Research on vulnerable participants is often contentious because of compromised autonomy and other logistics	Social justice considerations are primary. Working with vulnerable groups essential to understand implementation issues in these groups so that the intervention can reach them

^a^Developed from References [2, 6–13, 20, 43]

**Fig. 1:**
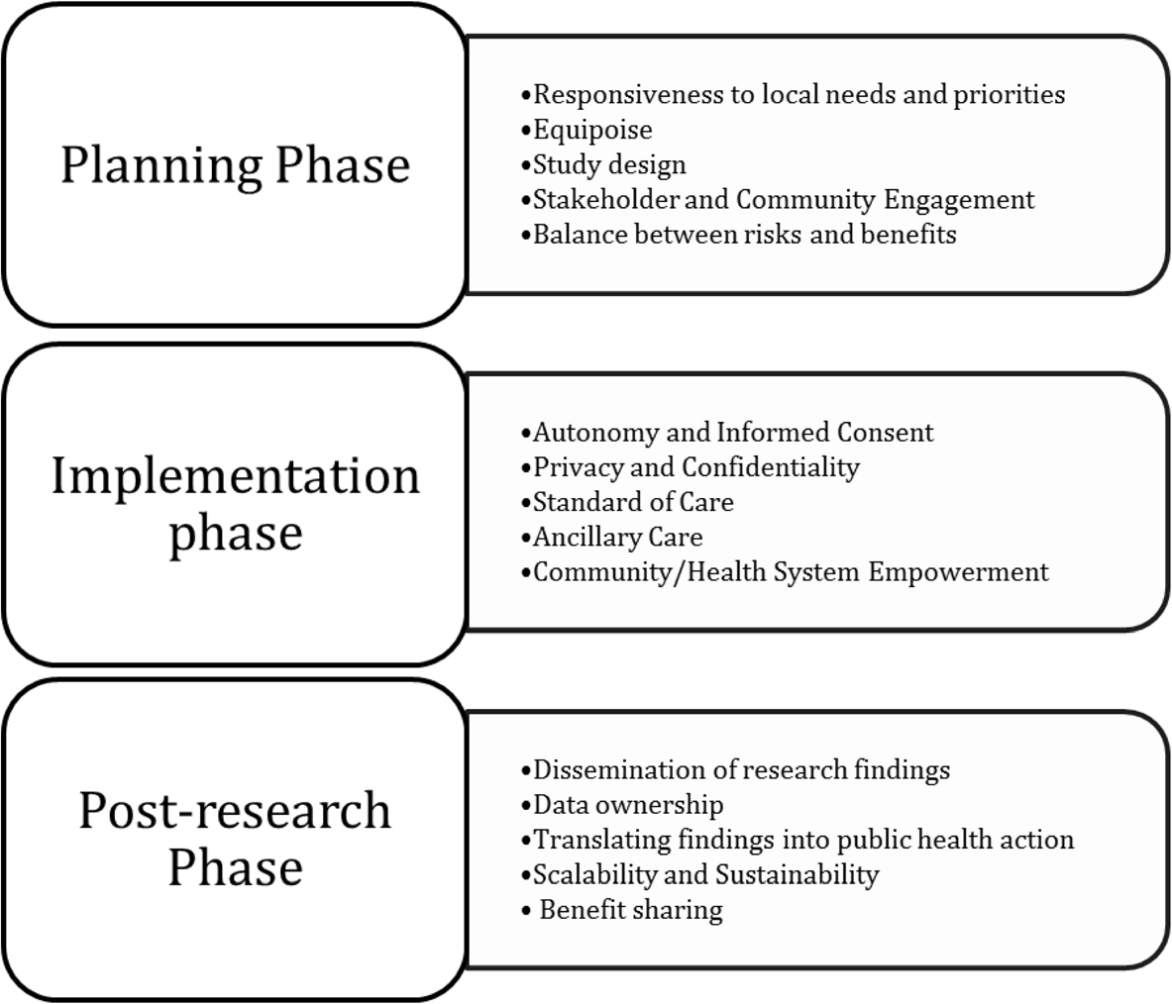
Ethical considerations in various phases of Implementation Research

Most researchers and research ethics committees are familiar with the ethical challenges posed by traditional clinical research, which emphasizes respect for individual autonomy and the importance of individual informed consent, beneficence, justice, and the necessity for clinical equipoise. Clinical research is usually carried out to answer a focused clinical question, under controlled circumstances, in a well-defined subject cohort with rigid inclusion and exclusion criteria. In IR, in contrast, because the goal is to generate knowledge that leads to wide-scale implementation of interventions in new contexts, the priority focus is different from that of clinical research [2, 6–13]. IR tends to occur in real-world circumstances to test the feasibility and effectiveness of an intervention in real-life situations and not under the controlled environs of clinical research, therefore the boundary between research and clinical care/public health practice can be quite blurred. The implementation ethics issues that arise in the context of IR may also be distinguished from those that arise in the context of programmatic implementation [14]. A priority in IR is broad inclusion of research subjects, specifically including vulnerable populations to optimize equity and justice in access to the intervention. An ultimate goal of IR is to generate knowledge that will be translated into health policy and health action, and therefore studies must be conducted with the vision of sustainable scale-up and roll-out of effective interventions. Although many ethical principles are common to biomedical research and IR, there may be some differences in the way these principles are applied. The key ethical issues relating to IR may be broadly divided into those that arise during the planning phase, the implementation phase, and the post-research phase. Although some ethical issues cut across multiple phases, for the purposes of this review we will discuss the issues as illustrated in Fig. 1. A case study is presented in Table 2 to illustrate the relevance of such ethical issues in the conduct of IR (case adapted from published experiences for the didactic purposes of this manuscript) [15].

**Table 2:** A case study illustrating the multiple ethical challenges arising in implementation research

Implementation research of strategies to improve vaccine coverage in children in nomadic populations
*Study description*. Country *X* had a low rate of vaccine coverage largely because a significant group of nomadic populations were not reached by the routine vaccination strategies. A basic needs assessment was performed among the nomadic populations and found their vaccine coverage rate to be very low. In addition, the assessment found that insufficient knowledge of the location of the nomadic populations, lack of logistical support and lack of community engagement in the vaccination drives were important reasons for poor coverage. In order to overcome these problems, an implementation research study was planned. Special Outreach Teams (SOTs) were trained and deployed to a selected sample of known nomadic groups. These SOTs were provided with all logistical requirements such as vehicles, ice boxes, vaccine stocks, and temperature logs. They were also trained to engage with the communities, to deliver the vaccinations to the children under five according to schedule and also to collect data on the existing level of vaccine coverage, numbers vaccinated, documentation of feasibility challenges, and costs. The SOTs coordinated their work with the routine health care workers in the communities where these nomadic groups were stationed at the time of contact. In addition, a small subsample of the nomadic groups were invited to participate in a mobile phone-based GPS tracking study to assess the feasibility and utility of locating the nomadic groups in real-time. Solar powered battery packs were provided to the key members of the community who held the GPS tracking mobile phone. Their GPS coordinates were relayed to the SOTs so that they can deliver their services effectively.
*Ethical issues*. This implementation research study brings out several important ethical considerations. There is an ethical imperative to engage and work with this special marginalized group in order to increase coverage of vaccination as it is the duty of the health system to protect and promote their health and also in order to more effectively protect the rest of the community with whom the nomadic groups will come in contact. Equipoise to justify the conduct of the study lies primarily in the uncertainty of how the intervention will be taken up and effective within this context as it is known that the vaccinations are effective from other populations. Despite this fact, however, effectiveness of the vaccine should be tracked in this new population as there may be modifiers of the effect, e.g., nutritional status etc. that may also require ancillary care. The findings of the study will help understand the feasibility and acceptability of the intervention among nomadic communities thus facilitating the implementation of the vaccination coverage campaign. Community engagement is a key ethical consideration in this context. Marginalized communities like these have inherent mistrust in health systems and community engagement helps build trust. Identification of appropriate gatekeepers of the community by appropriate selection process will facilitate both the informed consent process as well as representation of long-term voices of the communities. The tracking of the position of the nomadic groups for the sake of facilitating the vaccination process using the GPS tracking system is a significant intrusion into the privacy of the communities. This needs to be carefully weighed and balanced against the benefits of enhanced vaccine coverage and reduced child mortality because of that. Moreover, confidentiality of the GPS tracking data should be clearly maintained. Issues of sharing the GPS tracking position with local health system, other parties who may be interested to track them for other purposes etc. needs to be carefully deliberated. In addition, it is likely that many other health needs would be identified in these communities, raising the ethical issues associated with ancillary care responsibilities.

### Ethical concerns in the planning phase of IR

#### Responsiveness of IR

Problems addressed by IR must be of high local priority in order to justify the research [16]. One could argue that the requirement for responsiveness is greater from IR compared with clinical research as IR should address priority health needs whereas clinical research is concerned more with proof of principle, and wider application should be tested with subsequent IR. Conversely, when there is an available intervention for an important unmet health need in a community, there is an ethical imperative to conduct IR to try to address the implementation barriers. As such, IR studies are often commissioned by local health authorities. The knowledge of which problems are indeed local priorities relies on epidemiology and health data reporting, which may not always be optimal in resource-challenged environments. Engagement with local health experts and communities is therefore essential in the planning stages of IR to determine whether a health problem is indeed perceived to be a local priority. If a particular problem is not perceived to be a local health priority, the ethics of conducting IR becomes questionable [17].

#### Equipoise

Equipoise is an important ethical imperative in the conduct of research, it is required to justify any potential risk to research subjects. Clinical equipoise refers to the fact that investigators conducting a randomized controlled trial do not know in advance if an intervention is better than what it is being compared with. In IR, however, such clinical equipoise is generally not present (e.g., a medication is known to cure malaria), but instead, situational or contextual equipoise justifies the conduct of IR, i.e., there remains genuine doubt whether a new and untested package of interventions will work in a specific context [18]. To ethically justify IR, therefore, equipoise regarding the effectiveness of the implementation processes must be preserved.

#### Study design

A balanced discussion about study design is important before embarking on an IR study to weigh the ethical obligation to ensure scientific standards are maintained against the ethical demands of equity and justice [2, 19]. Though the randomized controlled trial is considered the gold standard in clinical research, health system strengthening interventions often do not lend themselves to such a design. Many different study designs, often with multidisciplinary involvement, have been used in IR, each raising particular ethical concerns [17]. Both qualitative and quantitative methods are used in IR and often within the same study. Examples of specific and different ethical considerations may arise with each method as outlined in Table 3. CRTs are often used in health systems research, but when a public health intervention is known to be effective, withholding the intervention from those randomized to the control arm is ethically problematic [4, 20, 21]. As a compromise, a stepped-wedge approach is sometimes justified to address this dilemma in CRTs, as this may mirror the real-world scale-up process [22]. In a stepped-wedge design, an intervention is delivered sequentially to groups of participants with the goal of ultimately including all participants, however in the early stages some groups do not receive the intervention and therefore are analogous to *controls*, which may pose an ethical challenge even if short-term because for that short period of time the participants are deprived of the proven intervention [23]. Alternatively, quasi-experimental designs where a control group may not be included may be ethically more acceptable in IR, but the scientific rigor and validity may be questioned [24]. The use of random allocation, without consideration of the specific needs and vulnerabilities of the participants, raises concerns of justice and equity. Other appropriate study designs for IR include pragmatic designs, hybrid and mixed methods designs, and open-label demonstration projects which may each have specific ethical issues that will require careful consideration at the planning stage [25–27]. Engagement with all stakeholders is crucial to develop the most effective and fair study design.

**Table 3:** Ethical issues relating to examples of implementation research designs^a^

IR design	Features	Example	Ethical concerns
Cluster randomized trials (group randomized, place-based, community wide intervention trials)	-Random allocation of groups or “clusters” to study arms and outcomes are measured in individual subjects and at community level	-Randomization of clusters of obstetrics unit staff to education on hand washing or usual practice, measurement of rates of puerperal sepsis in women delivering at study clinics	-Different units of intervention and outcomes measurement -Consent before and after randomization, whom to consent? -Choice of gatekeepers -No opt-out option within cluster -Risk: benefit balance -Ethics of randomization to known intervention, equipoise, -Identification of vulnerable groups
Effectiveness-implementation hybrid trials	-Assess both effectiveness and implementation strategy simultaneously -Identify intervention—implementation interactions	-Evaluate impact of ITN on reduction of malaria and assess robustness of availability and uptake of ITNs in the community	-The trade-off between the scientific rigor required for effectiveness assessment and the realistic contextual considerations required for implementation is an important ethical consideration
Mixed-methods research	-Use of both qualitative and quantitative methods -Understands various perspectives -Rationales: “participant enrichment”, “instrument validity”, implementation validity”, “meaning enhancement”	-Integration of HIV and TB management in single clinics—patient experience (qualitative) and adherence (quantitative)	-The trade-off between the scientific rigor required for quantitative methods and the realistic contextual considerations required for the qualitative component
Participatory action research	-Research question, design, and data collection in a participative manner by the research participants -“Bottom-up” approach	-Peer support groups to improve adherence to ARV in HIV + subjects	-There is a need for community engagement to ensure responsiveness, sustainability, and scalability
Pragmatic trials	-Effects of intervention in routine practice -Maximize variability of settings, practitioners, patients	-Introduction of community health workers for home management of malaria	-There may be concerns of standards of care and ancillary care, which in pragmatic conditions may be ethically debatable.
Quasi-experimental study	-Real-life conditions -With or without control group No randomization	-Open label demonstration project of effectiveness of self-reported use of pre-exposure prophylaxis for HIV	-There is a concern regarding scientific rigor of the research
Realist view	-Analysis of how and why an intervention works in a context combining theory and empirical evidence.	-Integration of traditional healers into home management of malaria strategies	-Community engagement is of utmost importance to retain cultural and contextual sensitivity

^a^Adapted from References [5, 17, 20, 21, 24, 26, 60]

#### Stakeholder and community engagement

The term *stakeholder* has numerous definitions, many of which are contextual [28]. Two general definitions are relevant in the context of IR: the first defines a stakeholder as a “person or group with an interest, involvement or investment in something” [29]; the second describes stakeholders as “people who will be affected by a project, or who can influence it, but who are not directly involved in doing the work” [30]. Key stakeholders in IR may include the government, policy-makers, public health functionaries, health care providers, health care managers, financing mechanisms, health care industry, and the community. Communities may include individuals on who interventions are planned, the broader community or social structures to which these individuals belong and the broader society to whom an intervention may eventually be rolled out. Communities and individuals with specific roles are important stakeholders in the research process. Meaningful engagement with stakeholders at all levels is crucial in IR, as a means to identify health priorities, to identify key participants, to communicate transparently and effectively about the goals, design, risks, benefits, and process of a proposed intervention, to gain trust and develop partnerships to enhance success of the study, and to gain feedback and identify unforeseen barriers that could be mitigated at the planning stage [31, 32]. Community engagement is a related but different concept where the members of the community who will benefit from or face the risk of the IR are actively consulted and engaged with, with the goal that they play an active partnership role throughout the IR process.

Stakeholder and community engagement are cross-cutting processes which must be carried out during the planning, implementation, and post-research phases of IR. Particular ethical underpinnings of engagement with policy-makers and health financers at the planning stage include determination that the intervention will address a local priority health need and to gain buy-in and commitment for the scale-up and sustainability of an effective intervention. Scale-up is considered by some to be an ethical corollary of IR. Important ethical goals for engagement with communities as partners in planning and design of IR include to determine acceptability of a proposed intervention, maximize uptake, ensure inclusion of vulnerable populations, establish accountability processes, and particularly when individual informed consent is not feasible due to the research design, to ensure individuals are aware of the rationale and opt-out possibilities. The process of decision-making in IR should consider the power differential between the researchers and the community and allow adequate representation of the research participants and the community at large [33–35].

Often community representatives are selected to facilitate communication between researchers and the community. The selection of community representatives must be an inclusive and fair process of democratic election or nomination, guided by the community itself, to ensure appropriate, acceptable, and comprehensive representation of all sectors of the community irrespective of class, race, gender, sexual orientation, or ethnicity and to avoid any potential conflicts of interest-specific individuals may have [33–36]. Engagement with disadvantaged and marginalized groups is imperative up-front and throughout the IR process to ensure acceptability and equitable participation in IR, and importantly, to identify any specific unanticipated barriers they may face and to develop strategies to mitigate further marginalization or stigmatization through the research [8, 37].

#### The balance between the risks and benefits

The clinical efficacy and safety of an intervention is generally known before IR is conducted. In clinical research, an individual participant can personally weigh the relative risks and benefits before giving their informed consent, and the risks and benefits are usually borne by the same individual. For example, in clinical research testing, the efficacy of a new vaccine, the benefit of personal protection, and the risk of side-effects are borne by the individuals who participate in the study. In IR, for example in mass drug administration interventions, the community may benefit from large-scale treatment of individuals, but an individual may experience side-effects from a medication they may not personally have required. In addition, the potential risks of an IR intervention may also result from the modality of implementation [2]. For example, a community wide public health screening campaign for sexually transmitted infections which had been successful in one low-income country may carry different risks of stigmatization, religious ostracism, and social discrimination if implemented in an underdeveloped and religiously orthodox country, leading to a different risk-benefit balance. The risks associated with IR may not always be obvious up-front as health systems are complex adaptive systems, and interactions between the components in the health system are not often clearly understood [38]. Diligent situational analysis must therefore be conducted during the planning phase of IR to identify potential risks before harm is done [19]. In addition, a particular feature of IR is that at times an intervention is implemented in one group, but the benefit may accrue to another group [2]. For example, IR studying the implementation and uptake prior to scale-up of a malaria transmission-blocking vaccine exposes vaccinated individuals to the risks of vaccination, but unless a large proportion of the community is vaccinated, the individuals vaccinated will protect others from malaria transmission, but will not be protected themselves. How to balance the risks experienced by one group against the benefits gained by another requires ethical deliberation and effective communication with the research participants. The ethical deliberation should be based on the solidarity principle and should be transparent, involving communities and all stakeholders [39]. To what extent individuals within a group should be exposed to risks for the benefit of others cannot be clearly defined, but it should be decided based on community and stakeholder consultations [2]. A line which cannot be crossed is knowingly exposing one group to harm or significant risk for the benefit of another.

### Ethical concerns in the implementation phase of IR

#### Autonomy and informed consent

A key principle driving the ethics of clinical research in humans is individual autonomy. In public health research autonomy has two dimensions, one concerns individual autonomy and the other concerns relational autonomy in the context of the community to which the individual and the health system belong [40]. Informed consent is the process through which a research participant can exercise their autonomy. In clinical research, a fully informed individual can determine whether or not they wish to freely participate in a study and can usually opt-out of the research at any stage. In IR, there may be difficulties in operationalizing informed consent [2, 20, 41, 42]. For example, an individual in a cluster in a CRT may not have the chance to decide and give consent to randomization as randomization happens at the cluster level. In the case of non-excludable cluster level, interventions such as environmental modifications, an individual may not be able to exercise a meaningful refusal to participate. In such situations in IR therefore there is a need to articulate informed consent differently from traditional individual consent in a clinical trial. At one end of the spectrum is a complete waiver of individual informed consent where the ethical risks are minimal, and the interventions are largely at a cluster level (i.e., no individual can opt-out), rendering refusal meaningless. For example, in an IR study of an ultraviolet wave system to provide safe drinking water to a population, the harms are considered minimal, and it is not possible for any individual participant in a cluster randomized to the intervention arm to easily opt-out of the study [43]. Such a waiver of consent does not however preclude the need for meaningful community engagement and provision of information. At the other end of the spectrum is the example where individuals have the opportunity to refuse participation in the research project, even though the intervention will occur at the cluster level. This could occur for example when public health professionals wish to test whether community health workers can be trained to provide injections in the community, and individuals have the option to refuse participation and visit the health facility to receive the injection from a nurse. Individual informed consent would be the norm in this case, even though consent for randomization cannot be provided by the individual and it is operationalized at the cluster level prior to individual contact [20, 43]. In the middle of the spectrum is dual consent from gatekeepers and individuals. Community agreement relies on the identification of an appropriate gatekeeper, who should have a keen interest in the welfare of the community and represents the community in a fair manner [36]. There are several challenges in selection of the gatekeeper. In traditional communities where collective decision-making is practiced, selection of a gatekeeper may not be problematic. But in more complex societies, or more complex studies, selection of one voice to represent the community is often challenging. The community leader may not be the most appropriate person to make decisions on whether a community or its members should participate in a study or not. For example, an elderly male village leader may not be an appropriate gatekeeper to consent to an IR intervention on pregnant women in his community. Selecting community representatives fairly requires inclusion of a variety of representative stakeholders, especially those from the target groups, and ensuring transparency of the process [20, 36]. The agreement of the gatekeeper, however, cannot replace individual consent or assent where relevant as discussed above [4]. Ultimately, it is important that proper ethical oversight is in place through Institutional Ethics Committees to ensure that the appropriate informed consent process is followed, maximally respecting autonomy of individuals in the study [44].

Challenges in operationalizing informed consent in the context of IR also include whether the beneficiaries are individuals or populations, and appropriate identification of who the actual research participants are [18]. For example, when implementing a taxi voucher system to increase the rates of institutional deliveries and reduce maternal mortality, should consent be obtained from the pregnant women, the taxi drivers, or the health care workers whose performance will also be evaluated? The Ottawa statement on ethical conduct of cluster randomized controlled trials define a research participant as: the intended recipients of the experimental or control intervention; the direct targets of experimental or control environmental alterations; persons with whom researchers interact to collect their data; persons whose identifiable private information is accessible to the researcher for collection of their data [21]. As such, in the example above, patients and health care workers should provide consent, but whether this should extend to the taxi drivers is questionable and may be difficult to operationalize. A further important ethical issue in the informed consent process is the extent of information to be revealed to the participants in the intervention and control arms, where applicable. In IR, especially when behaviour change interventions are being studied, knowledge of the intervention itself may change the outcomes and implementation process. There is, therefore, often the need to conceal some information about the intervention. The ethical justification for this is debatable, and it must be balanced against the risks/benefits and the potential impact on study validity as discussed in the CIOMS guidelines [45]. The informed consent process in IR therefore may be quite different from that in clinical research and requires thorough consideration to ensure optimal ethical conduct of IR.

#### Privacy and confidentiality

Particular issues relating to privacy and confidentiality in IR relate to the fact that IR often requires that facility level data on patient outcomes be available or that individual level data from facility health records be obtained. For example, if a public health intervention is implemented to regulate institutional deliveries and improve the quality of skilled institutional deliveries, there may be interventions at the health facility level, but confidentiality restrictions on access to data from women who deliver in the facilities may hamper effectiveness analyses of the health system impact [19]. In such cases, the data that is obtained from facilities should either be anonymized, or the individuals about whom data is being sought should provide consent for their data to be reviewed by the researchers. Where such consent is not possible, it is the responsibility of the researcher to obtain a waiver of consent from the respective ethics board, and put in place mechanisms to ensure that the confidentiality of the patient information is respected. A proactive strategy of informing patients about potential data collection for research and quality improvement purposes up-front, but reassuring them about privacy and confidentiality could also serve strengthen the patient-researcher partnership and build trust [46].

#### Standard of care or prevention

There are two approaches to decide on standard of care or prevention to be given to a control group [47–49]. One approach is to allocate the *local de facto* existing standard, which in some situations may be grossly insufficient, making it ethically unacceptable based on justice and fairness principles. For example, in an IR trying to study prevention of mother to child transmission of HIV in a country where routine anti retroviral therapy is not available, the *local de facto* standard of care is no treatment. Having a placebo control arm in the study is not acceptable in spite of the *local de facto* care being no treatment, because an effective treatment which reduces transmission is available and should be accessible to the mothers. The second approach is to provide the *local de jure* standard of care or prevention, which is agreed upon by public health experts of that region and is acceptable to the community. This approach may still be unfair in that this standard may be unsustainable for the local health system after the IR is completed. For example, in a public health behaviour change implementation study focused on hand washing among schoolchildren, the intervention group receives a school-based lunchtime hand washing program, and the control group receives soap and water in all schools, but without any emphasis on hand washing before eating. In this case, the standard of care is provision of hygiene tools, and the intervention is emphasizing the use of these tools. In this context, allowing the control group to have no intervention can be considered ethical. The consideration of standards of care or prevention may therefore identify new gaps as targets for future IR.

#### Ancillary care

Ancillary care refers to the identification of problems that may contribute to ill-health that are beyond the scope of the study in question, for example, researchers studying home management of malaria may come across household members with other diseases needing attention [11, 50, 51]. Sometimes ancillary care responsibilities can be foreseen at the design stage and at others they are encountered only during the conduct of the IR. Ancillary care obligations are present when the need is serious in terms of severity or urgency or both and when there is a possibility of provision of care within the scope of the research [11]. For example, in the school-based hand washing behaviour change IR study, uninterrupted tap water supply may be lacking and this is an ancillary care requirement. However, this example illustrates that it may not be realistic to expect implementation researchers to assume all ancillary care responsibilities. Researchers may not have the expertise to provide the ancillary care; the provision of the care may be costly or may require system-level interventions. The researchers must, however, establish process of accountability for ancillary care need identified through the research, determine which needs may realistically fall within the scope of responsibility of the researchers, and proactively engage with the local government or non-governmental organizations during the planning and conduct phases of IR to identify who will be able to meet other needs [52].

#### Research capacity and health system strengthening

Well conducted IR should lead to strengthening of research capacity of the local institutions as well as individuals’ capacity to conduct research in settings where such capacity is weak [53]. Research capacity strengthening can range from creating a trained workforce of researchers and research volunteers up to training and capacity building of research experts and infrastructure to permit independent conduct of locally responsive IR in the future. Based on the need in the area where the IR is being conducted, appropriate research capacity strengthening should be facilitated. This can be facilitated by appropriate stakeholder engagement ensuring commitment by donors and governments to build sustainable research capacity. Not only is it important for the IR to strengthen local research capacity, it should also strengthen the health system within which it is conducted. For example, true partnership in an implementation research study of rapid diagnosis of tuberculosis resistance should build sustainable infrastructure in technology required, expertise to run and maintain the technology, strengthen the local health information system to track data acquired, train local researchers in design, conduct, analysis and reporting of study findings as well as participation in post-intervention scale-up, thereby strengthening the local research and health system capacity. IR projects focusing on specific health gaps may, however, create vertical program structures within the health system which may be disempowering to the system through inefficient resource utilization [13]. It is ethically important that the conduct of IR should focus on horizontal integration of public health interventions into the health system such that a project empowering any component of the health system may have positive repercussions for the entire system. Strengthening the capacity to translate research findings into health policy is a specific imperative in IR and must be a component of all phases of the IR process [53].

### Ethical concerns in the post-research phase of implementation research

#### Dissemination of research findings

Given the important public health impact of IR, there is an ethical obligation to disseminate the research findings widely, including feeding back to the communities and stakeholders who participated in the research [54, 55]. If an implementation strategy had a negative or positive impact in a certain context, either finding may be important for researchers planning similar interventions elsewhere. Therefore, irrespective of the results, the findings of the IR should be disseminated. Furthermore, resource utilization globally could be enhanced by an imperative for dissemination of IR findings, as once an intervention has been tested in many different local contexts, its findings may be presumed to be generalizable and obviate the need for new IR studies and delays in scaling-up of the intervention in new contexts.

#### Data ownership and sharing

In case of donor or sponsor-driven IR, data is often owned donor, who may regulate and restrict further handling of the data. Data ownership should be fairly negotiated through transparent stakeholder engagement in the planning phase of IR, and ethical oversight of the data ownership process is required to ensure appropriate access to the research findings by the relevant stakeholders post-study, including the local researchers and communities when appropriate, to maximize the utility of the knowledge generated. It may be acceptable for researchers or donors to *own* data without further responsibility. However, given the policy and public health implications of IR and the necessity of trust especially with the communities, there may be a responsibility for data sharing which should also be negotiated up-front, considering the important implications of protecting privacy and confidentiality as well as to allow strengthening of local research capacity.

#### Translating findings into public health action

Due to the inherent nature of IR, there is an ethical obligation for IR findings to be used to inform effective and equitable public health action. This necessitates timely consideration and uptake as relevant of the IR findings into public health policy and practice. Potential barriers to translating knowledge into action include lack of prior consultation with policy-makers, lack of funding, weak health systems, poor communication of findings by researchers to policy-makers, and absence of a culture of evidence-based decision-making among others [53]. Therefore, in order to translate the research into public health action, implementation researchers should engage with policy-makers and health system officials, important stakeholders in IR, upfront to ensure commitment to sustainability should the intervention prove successful, and must communicate their research findings rapidly, clearly, and concisely to engage and inform policy-makers in a timely fashion. Researchers should also propose actionable suggestions based on the research findings to facilitate uptake and scale-up of successful interventions. Barriers identified during IR may require further study to develop strategies to overcome them. Effective communication between researchers and policy-makers, as well as education of the public are important social justice obligations in IR, ensuring that expectations raised during the research are met, and those who participated as control subjects gain access to interventions withheld from them during the study.

#### Scalability and sustainability

Scalability and sustainability are important ethical considerations at both planning and post-study phases, as ultimately these are the goals of IR [13, 56]. The duty to ensure sustainability post-study cannot only lie with the researchers. Multiple stakeholders must come together to promote this goal which requires ongoing stakeholder engagement throughout the IR process. The researchers should ensure through effective engagement during the planning and conduct stages of IR that the non-research stakeholders such as policy-makers, local providers, and health system officials remain committed to sustaining implementation of an intervention if found to be effective. If access to a proposed public health intervention cannot be ensured for a community after the IR, it may not be ethical to carry out such a research activity. If specific interventions are provided during IR without a plan for sustainability, this could lead to exacerbation of inequity and harmful effects to the community as well as loss of trust in the health system.

#### Benefit sharing

Irrespective of the context in which IR is conducted, LMIC or developed countries, there is an ethical obligation to share benefits of the IR with the community [57]. There are various classifications of the benefits that can be achieved as a result of conduct of the IR. The benefits may be direct as a result of the intervention being studied, or indirect and not related to the intervention per se. The benefits may accrue to individual participants or to the community at large. For example, IR may be conducted in communities where the local health system is weak, therefore success of an intervention may result in introduction of a new intervention that was effective in the local context, providing individual benefit. In addition, the IR likely identified and overcame barriers which would have contributed to some strengthening of the local health system that would have a broader impact. IR researchers can facilitate sharing of direct benefits by advocating for sustainable translation of research findings into action, and sharing of indirect benefits through building research capacity and health system strengthening. The unique nature of IR where the individuals who bear the risks of the IR are not always the ones who enjoy the benefits is a challenge as discussed above. Optimal benefit sharing can be promoted through proper pre-IR planning and ethical conduct. Community and stakeholder engagement plays an important role in achieving benefit sharing as when adequately informed they can advocate for access to proven benefits. Benefit sharing has important social justice implications, and it is the obligation of the researcher to achieve a balance of risks and benefits to both individuals and communities [58, 59].

## Conclusion

Putting public health evidence into practice in specific populations requires the generation of knowledge about the feasibility of public health interventions within a specific context, the relative harms and benefits, how an intervention is taken up, whether it reaches the most vulnerable populations, and the logistics of the implementation process. IR aims to generate this knowledge with the goal of enhancing health system performance while upholding fairness and justice in the reach of the intervention to all parts of the community. The ethical principles pertaining to IR are not unique to IR, but may require adaptation in application given the particularities of IR. The stakes of IR are high because of the research contexts within fragile health systems, the large numbers of subjects involved and the reduced ability to predict outcomes and consequences as compared to clinical research [8]. Awareness of the ethical challenges relating to IR is important throughout the planning, implementation, and post-study phases of the research not only to ensure studies are conducted appropriately and that results are maximally useful, but is also important for ethics review committees and institutional review boards to provide appropriate and insightful review of IR projects.

This paper emerged out of the development of the Ethics in Implementation Research Toolkit. Through the consultation process, an important need was identified to clarify the differences in the application of research ethical principles between clinical research and IR, both to guide researchers in planning and conduct of IR and to facilitate review of IR proposals by research ethics committees. As such, this paper complements the Implementation Research Toolkit (http://www.who.int/tdr/publications/topics/ir-toolkit/en/) and the Framework for Operations and Implementation Research in health and Disease Control Programs (http://www.who.int/hiv/pub/operational/or_framework.pdf). It is hoped that this paper will generate discussion in further refining roles and obligations of implementation researchers in low resources settings and in further defining the obligations of policy-makers and funders in committing to long-term sustainability of successful interventions.

## Footnotes

1 PAR is a community-based approach to research that emphasizes community participation in research design, data collection, analysis, and interpretation. PAR tries to understand situations and phenomena by changing them and making observations. The experimentation is grounded in community wisdom and experience.

2 Effectiveness implementation hybrid designs include simultaneous testing of clinical effectiveness and effectiveness of the method of implementation. It may be of three types: (1) primarily doing an effectiveness study while at the same time gathering data on implementation, (2) primarily doing an implementation study while simultaneously gather data on effectiveness, or (3) dual testing of both effectiveness and implementation strategies.
